# Suppressive Effects of an Inhibitor Composition on Skin Ulceration and Transcriptomic Analysis in the Sea Cucumber *Apostichopus japonicus* Exposed to No. 0 Diesel Oil

**DOI:** 10.3390/biology15060482

**Published:** 2026-03-18

**Authors:** Xiaonan Li, Yajie Deng, Shufeng Li, Haoran Xiao, Fenglin Tian, Qi Ye, Lingshu Han, Chong Zhao, Jun Ding

**Affiliations:** 1 Liaoning Provincial Key Laboratory of Northern Aquatic Germplasm Resources and Genetics and Breeding, Dalian Ocean University, Dalian 116023, China; lixiaonan0724@163.com (X.L.); dengyajie0220@163.com (Y.D.); lishufeng0919@163.com (S.L.); xiaohr1218@163.com (H.X.); tfl9904@163.com (F.T.); yeeqii1999@163.com (Q.Y.); hanlingshu@dlou.edu.cn (L.H.); chongzhao@dlou.edu.cn (C.Z.); 2 Key Laboratory of Mariculture & Stock Enhancement in North China’s Sea Ministry of Agriculture and Rural Affairs, Dalian Ocean University, Dalian 116023, China

**Keywords:** no. 0 diesel oil, *Apostichopus japonicus*, skin ulceration, inhibitor composition, transcriptomic

## Abstract

No. 0 diesel oil may pose a serious threat to sea cucumber (*Apostichopus japonicus*) aquaculture by inducing skin ulceration. This study aimed to evaluate the protective efficacy and mechanism of a previously developed inhibitor composition against diesel-induced injury. The inhibitor composition significantly alleviated skin ulceration in the experimental group (Eg), reducing the lesion area to 14.44 ± 1.79% after 96 h, compared to 33.19 ± 2.94% in the diesel-exposed control group (Cg) (*p* < 0.05). It effectively suppressed the overactivation of autolytic enzymes (cathepsin L and B) while enhancing the activities of acetylcholinesterase, superoxide dismutase, and catalase. Transcriptomic profiling revealed 3137 differentially expressed genes, with functional enrichment in pathways related to Notch signaling, ECM–receptor interaction, glycosaminoglycan biosynthesis, and detoxification. The upregulation of genes such as *HES-C*, *CYP1A1*, *GST*, and *UGT* may be linked to the regulation of apoptosis inhibition, xenobiotic metabolism, and antioxidant defense. Furthermore, enhanced expression of NAD kinase and PNLIPRP may indicate a potential strengthening of energy metabolism and lipid utilization during stress adaptation. This study suggests that the inhibitor composition may exert a multi-level protective effect against diesel-induced injury by coordinating tissue repair, oxidative balance, and detoxification processes, offering a potential strategy to mitigate pollution impacts in sea cucumber aquaculture.

## Simple Summary

Marine diesel oil pollution poses a serious threat to sea cucumber aquaculture by causing skin ulceration and physical damage. This study investigated the protective effects and mechanisms of an inhibitor composition under diesel stress. The results showed that the inhibitor significantly reduced ulceration area, suppressed the overactivation of autolytic enzymes (cathepsin L and B), and enhanced antioxidant and neural function-related enzymes. Transcriptomic analysis revealed that the protective mechanism involves coordinated regulation of tissue repair, detoxification, and antioxidant pathways. This research offers a potential strategy to mitigate diesel pollution impacts in aquaculture, supporting marine environmental protection and sustainable fishery development.

## 1. Introduction

In recent years, marine oil spill incidents have occurred more frequently due to increasing marine transportation, oil and gas exploitation, and pipeline operations [1]. Oil contamination has become a severe environmental stressor in coastal ecosystems [2]. In China’s coastal waters, petroleum hydrocarbons are widely detected in seawater, sediments, and biota, with concentrations in regions like the Pearl River estuary and Bohai Bay showing concerning increases and even exceeding safety benchmarks in sediments and shellfish [3]. Specific incidents, such as in 2010 Dalian Xingang Port spill that released approximately 1500 tons of crude oil, have caused direct and severe economic losses to local aquaculture industries [4]. Among prevalent petroleum pollutants, No. 0 diesel oil, commonly used as ship and machinery fuel, represents a major contaminant in marine environments [5]. Its persistence and toxicity pose a particular threat to benthic organisms.

The sea cucumber (*Apostichopus japonicus*), an economically vital echinoderm prized for its nutritional and bioactive properties [6], inhabits intertidal and shallow benthic zones [7,8], maintaining close contact with sediments. This ecology renders it highly vulnerable to diesel contamination from spills or chronic pollution. The scientific rationale linking diesel exposure to skin ulceration lies in the triggering of a localized “autolytic cascade”. Under conditions of high diesel oil contamination, the total antioxidant capacity of sea cucumbers significantly decreases, indicating suppression of their antioxidant defense system [9]. Previous studies have established that exposure to No. 0 diesel oil induces oxidative stress in *A. japonicus*, manifested by reduced activities of key antioxidant enzymes such as superoxide dismutase (SOD) and catalase (CAT) [10,11]. This oxidative imbalance further triggers a series of cellular events, including lipid peroxidation and the aberrant activation of endogenous autolytic enzymes (such as cathepsins and matrix metalloproteinases). These enzymes, which normally regulate tissue turnover, begin to degrade the extracellular matrix (ECM) and cellular structures indiscriminately, culminating in the manifestation of skin ulceration. [12]. This process not only threatens sea cucumber health and survival but also undermines the sustainability of aquaculture. Given the severe ecological and economic impacts of this tissue degradation, it is highly necessary to develop targeted chemical intervention strategies to mitigate the skin ulceration process and protect sea cucumber populations from environmental stressors.

In our previous study, our research group developed a chemical intervention composition consisting of three inhibitors: Nafamostat mesylate (FUT-175, a broad-spectrum serine protease inhibitor), Ilomastat (GM6001, a broad-spectrum matrix metalloproteinase inhibitor), and Emricasan (IDN-6556, a caspase-3 inhibitor) [13]. The design of this composition specifically targets the key executioners of the autolytic process: Nafamostat and Ilomastat inhibit the proteases responsible for tissue degradation, while Emricasan blocks the apoptosis triggered by environmental stress. This composition was demonstrated to significantly inhibit the *A. japonicus* skin ulceration phenomenon induced by UVA irradiation and *Vibrio splendidus* exposure [14]. While these results are promising, it remains crucial to elucidate whether the inhibitor composition exerts similar protective efficacy against chemical pollutants like No. 0 diesel oil, which may initiate damage through distinct toxicological pathways. Environmental stress is often mediated through mechanisms such as oxidative stress, disruption of membrane homeostasis, and imbalances in key signaling pathways, thereby affecting tissue remodeling and the skin ulceration process. Hence, investigating the inhibitory effects and underlying mechanisms of this inhibitor composition under No. 0 diesel oil pollution is of great importance for revealing the molecular mechanisms by which it regulates the skin ulceration process in *A. japonicus*.

Based on the above findings, this study aimed to investigate the inhibitory effects and molecular mechanisms of this inhibitor composition against skin ulceration in *A. japonicus* under No. 0 diesel oil stress. A comprehensive analysis integrating phenotypic observation, enzymatic activity assays, and transcriptomic profiling was conducted to systematically evaluate the protective efficacy of the inhibitors.

## 2. Materials and Methods

### 2.1. Sea Cucumbers

The *A. japonicus* used in this study were juveniles collected from Dalian, China. Their average initial body weight was 20 g ± 5 g. These individuals were at a pre-sexual maturity stage, following the size criteria for juveniles in this species [15]. A total of 36 *A. japonicus* were utilized in this study, with 12 individuals assigned to each group. Sampling was conducted at four time points with three independent biological replicates (*n* = 3) for each analysis. Prior to the study, they underwent a two-week acclimation period under controlled conditions, including a water temperature of 16 ± 1 °C, salinity of 31 ± 1‰, and pH of 8.0 ± 0.3. The *A. japonicus* were fed once daily at 14:00 with 3% of their body weight. To maintain water quality, the tank bottoms were cleaned daily, and any uneaten food and waste were promptly removed.

### 2.2. Experimental Design

At the beginning of the experiment, healthy *A. japonicus* individuals were randomly assigned to three groups: the experimental group (Eg), the control group (Cg), and the blank group (Wg). Each group comprised 12 individuals distributed among three replicate glass tanks, with 4 individuals per tank. Given that diesel oil and seawater are immiscible, continuous 24-h aeration was provided via air stones in each tank to maintain a consistent and well-mixed exposure environment. This setup created constant upward turbulence to simulate natural wave action, ensuring the diesel oil remained in a stable suspension. To facilitate direct contact between the sea cucumbers and the resulting surface oil film during these water fluctuations, each tank was filled with 4 L of natural seawater, which also ensured an appropriate loading rate for the juvenile individuals. The experimental design was based on the method outlined by Liu et al. [16].

**Figure 1:**
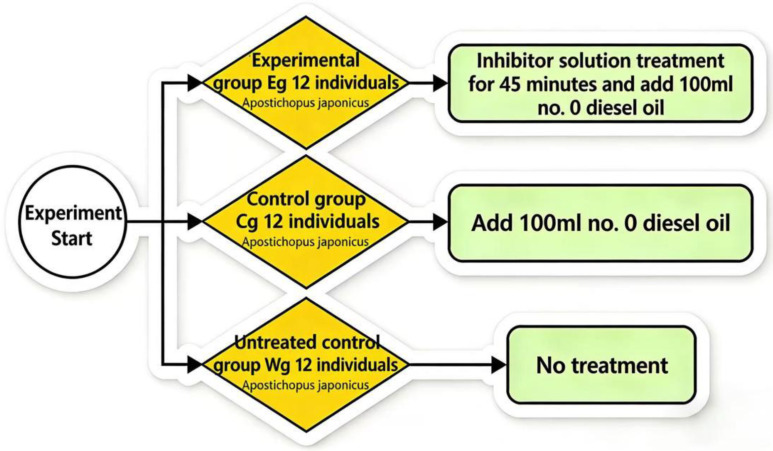
Schematic diagram of the treatments for the three groups of *A. japonicus*.

Before the experiment, group Eg was treated by immersion for 45 min in a chemical intervention composition consisting of three specific inhibitors: Nafamostat mesylate (FUT-175), Ilomastat (GM6001), and Emricasan (IDN-6556), whereas group Cg received no treatment. Subsequently, 100 mL of No. 0 diesel oil was added to each tank of both group Eg and group Cg to establish the diesel-pollution exposure model, whereas group Wg was left untreated (Figure 1). Throughout the experiment, photographs were taken daily to record morphological changes, and the behavioral status and physiological responses of *A. japonicus* were continuously monitored. During the 96 h experimental period, body wall tissues were dissected and collected every 24 h (at 24, 48, 72, and 96 h). At each time point, three individuals (one per replicate tank) were randomly selected from each group and were not returned after sampling. This ensured that each analysis was based on three independent biological replicates (*n* = 3) that had not been previously disturbed. 

### 2.3. Evaluation of Skin Ulceration Severity in A. japonicus

**Figure 2:**
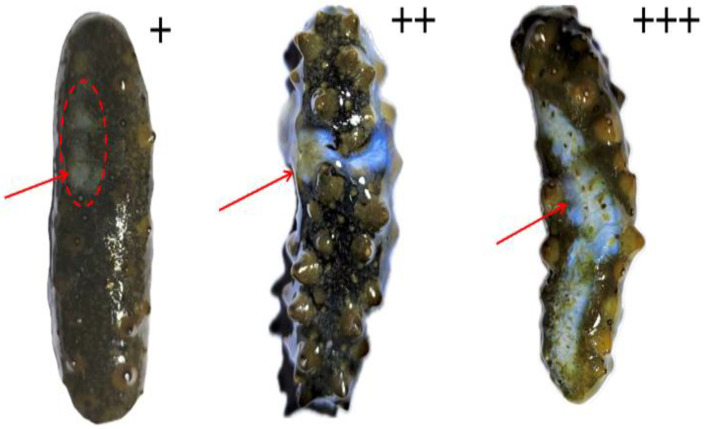
Representative images showing different degrees of skin ulceration area in *A. japonicus.* 1–10% of the skin is affected: +; 10–30% of the skin is affected: ++; 30–50% of the skin is affected: +++.

High-resolution photographs of the ulcerated surfaces of *A. japonicus* were acquired with a Canon EOS R5 (Canon Inc., Tokyo, Japan), and the posture of each specimen was standardized to ensure cross-sample consistency (Appendix A). Contrast was then adjusted in Adobe Photoshop 2021 (Adobe Inc., San Jose, CA, USA) to improve discrimination between degenerated and healthy tissues. Quantification was performed in ImageJ (v1.8.0; National Institutes of Health, Bethesda, MD, USA) [17]; each image was first converted to 8-bit grayscale, after which the “Threshold” function was applied to isolate regions of skin ulceration. The segmented areas were measured automatically by the software, and the resulting values were recorded. From the same images, the apparent body surface area of each animal was estimated, and the ulcerated area was expressed as a percentage of this surface area. This percentage represented the extent of skin ulceration induced by No. 0 diesel oil exposure. Severity was coded as “−” for < 1% ulceration, “+” for 1–10%, “++” for 10–30%, and “+++” for 30–50% (Figure 2) [14].

### 2.4. Measurement of Autolysis-Related Enzyme and Antioxidant Enzyme Activities

All experimental procedures were carried out strictly in accordance with the manufacturers’ instructions; each experiment was performed in triplicate (*n* = 3). Autolytic enzymes were extracted following the method of Zhu and Han [18]. Body wall tissues were homogenized in phosphate-buffered saline (0.1 mol/L, pH 6.3) using a Servicebio KZ-5F-3D homogenizer (Servicebio, Wuhan, China). The homogenate was then subjected to ammonium sulphate precipitation (20–70% saturation) and subsequent dialysis. All centrifugation steps were performed at 10,000× *g* and 4 °C using a Cence H1750R high-speed refrigerated centrifuge (Cence, Changsha, China). The resulting enzyme fraction was purified by Sephadex G-100 (Cytiva, Uppsala, Sweden) column chromatography. Enzyme activity was evaluated using a casein hydrolysis assay. Activities of acetylcholinesterase (AChE), cathepsin L (CL), cathepsin B (CB), superoxide dismutase (SOD), and catalase (CAT) were measured with commercial kits from Nanjing Jiancheng Bioengineering Institute (Nanjing, China): AChE (A024-1-1), CL (H635-1-2), CB (H440-1-2), CAT (A007-1-1), and SOD (A001-3-2). The absorbance was determined using a Multiskan FC microplate reader (Thermo Fisher Scientific, Waltham, MA, USA).

### 2.5. RNA Extraction and Library Construction

Body wall tissues from *A. japonicus* (*n* = 3 biological replicates per group, each containing pooled tissues from 3 individuals) were snap-frozen in liquid nitrogen. Library preparation and sequencing services were provided by Lianchuan Biotech (Hangzhou, China) following the manufacturer’s protocols. Total RNA was extracted using Trizol reagent (Thermo Fisher, 15596018) according to the manufacturer’s protocol. The quantity and purity of the RNA were assessed using the Thermo Fisher Qubit 3.0 (Thermo Fisher, Q33216) and the Agilent 5300 Fragment Analyzer (Agilent, Santa Clara, CA, USA, M5311AA). High-quality RNA samples (RIN > 7.0) were used for library construction. After RNA extraction, 2 μg of total RNA was purified using mRNA Capture Beads 2.0 (Yeasen, Cat. 12629ES, Shanghai, China) through two rounds of purification. The purified mRNA was then fragmented into short pieces using magnesium ions at 94 °C. The cleaved RNA fragments were reverse-transcribed into first-strand cDNA using reverse transcriptase. Second-strand DNA was synthesized using *E. coli* DNA polymerase I, RNase H, and dUTP Solution (Yeasen, Cat. 12340ES97). An A-tail was added to the blunt ends of each strand to prepare for adapter ligation. Dual-index adapters were ligated to the fragmented cDNA, and the ligated products were amplified by PCR with the following conditions: initial denaturation at 98 °C for 1 min, followed by 14 cycles of denaturation at 98 °C for 10 s, annealing at 60 °C for 30 s, and extension at 72 °C for 30 s, with a final extension at 72 °C for 5 min. The average insert size of the final cDNA library was 400 ± 50 bp. The PCR products were purified using Hieff NGS DNA Selection Beads (Yeasen, Cat. 12601ES75). Finally, the libraries were sequenced on an Illumina Novaseq™ X Plus platform (LC-Bio Technology Co., Ltd., Hangzhou, China) with 2 × 150 bp paired-end sequencing (PE150), following the manufacturer’s recommended protocol.

### 2.6. Data Quality Control and Genome Mapping

Sequencing outputs were cleaned by removing adapters and low-confidence bases, producing high-quality reads that were used for all later analyses. Standard QC indices (Q20, Q30, GC content) were derived, and FastQC v0.11.9 was applied to evaluate library quality; a Q30 proportion > 90% indicated a base-calling error probability of ~0.1% or lower. GC-content distributions were examined relative to the reference genome. Processed reads were aligned to the *Apostichopus japonicus* reference assembly obtained from Figshare (URL: https://figshare.com/articles/dataset/The_genome_annotation_files_of_Apostichopus_japonicus/22140020, accessed on 20 October 2025) using HISAT2 v2.2.1 with default parameters; per-sample alignment rates exceeded 85%.

### 2.7. Transcriptomic Analysis

Data obtained at 72 h were used for analysis. Read counts were normalized using DESeq2 v1.34.0 with default parameters. Raw read counts were processed in DESeq2 (v1.34.0) using default options to obtain size-factor-normalized values. Gene-wise differential expression was tested with the Wald statistic, and *p*-values were adjusted by the Benjamini–Hochberg false discovery rate (FDR) method. After DESeq2’s independent filtering, genes meeting FDR < 0.05 and |log2 fold change| > 1 were retained as differentially expressed. A volcano plot was generated to summarize effect sizes versus significance. KEGG pathway over-representation was assessed with clusterProfiler (v4.4.4) using Fisher’s exact test with FDR control, restricting gene sets to 5–2000 members [19].

### 2.8. qRT-PCR Validation of Transcriptomic Data

For validation, we quantified nine DEGs representing high, moderate, and low fold changes by qPCR. Gene-specific primers were generated with Primer Premier v5.0 (Table 1), and *n* = 3 biological replicates were analyzed per group. RNA was extracted with Promega’s SV kit (Z3100, Promega, Madison, WI, USA) and treated on-column with DNase I. Expression levels were derived via 2^−ΔΔCt^ with efficiency correction, normalized to NADH and a designated calibrator. All primers exhibited amplification efficiencies above 90% from standard-curve assessments. geNorm supported NADH stability (M < 0.5). PCRs (20 μL) comprised 1× SYBR Green Master Mix, with a program of 95 °C 10 min followed by 40× (95 °C 15 s, 60 °C 1 min).

### 2.9. Data Analysis

Data were summarized as mean ± SD from three independent replicates (*n* = 3). Analyses and plotting were performed in OriginPro 8.5, GraphPad Prism 8.0, SPSS 27.0, and R 4.3.1. To determine appropriate statistical procedures, we first evaluated normality and tested for homogeneity of variance. Both assumptions were satisfied across all datasets. Accordingly, two-way ANOVA was performed, followed by Tukey’s HSD for post hoc multiple comparisons. Differential expression for RNA-seq data was evaluated with DESeq2 using the Wald test and Benjamini–Hochberg adjustment; genes with adjusted *p* (FDR) < 0.05 were considered significant. All RNA clean data were submitted to the Short Read Archive (SRA) Sequence Database at the National Center for Biotechnology Information (NCBI) (Accession No. PRJNA1344652).

## 3. Results

### 3.1. Skin Ulceration Area Ratio of A. japonicus Induced by No. 0 Diesel Oil

As shown in Table 2, the skin ulceration area ratio of *A. japonicus* in group Eg was 14.44 ± 1.79% at 96 h, whereas that of group Cg reached 33.19 ± 2.94%. At all time points except 24 h, the ulceration area in group Eg was significantly lower than that in group Cg (*p* < 0.05). Exposure to No. 0 diesel oil significantly induces skin ulceration in *A. japonicus*, with the ulceration area increasing continuously with prolonged exposure. The inhibitor composition can significantly suppress this ulceration process, and the inhibitory effect is statistically significant at all time points except 24 h.

### 3.2. Effects of the Inhibitor Composition on Enzyme Activity Changes in the Body Wall of A. japonicus Induced by No. 0 Diesel Oil

#### 3.2.1. Autolytic Enzyme Activity

**Figure 3:**
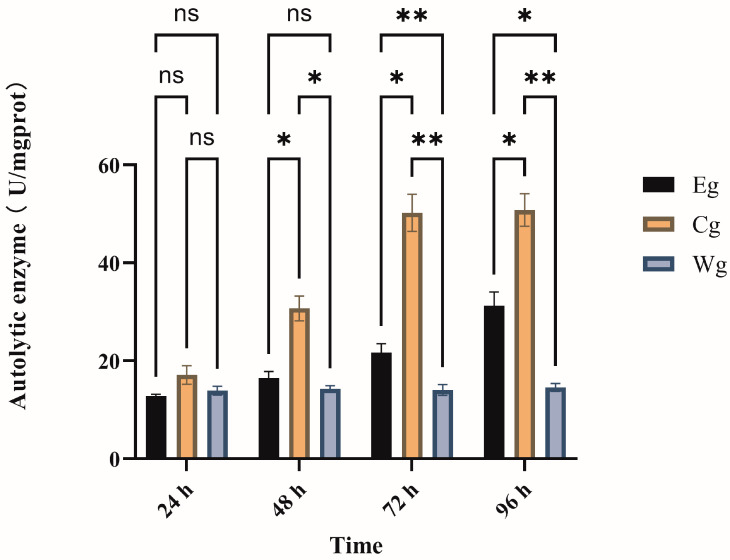
Effects of the inhibitor composition on autolytic enzyme activity in the body wall of *A. japonicus* under No. 0 diesel oil exposure. Note: “ns” indicates no significant difference. Significant differences are indicated by asterisks: * for (*p* < 0.05), ** for (*p* < 0.01).

As shown in Figure 3, two-way ANOVA revealed significant effects of both treatment and exposure time, as well as a significant interaction between these two factors (*p* < 0.05), on autolytic enzyme activity. In group Cg, autolytic enzyme activity increased sharply and continuously from 24 h to 96 h. In contrast, group Eg showed only a mild and gradual increase over the same period. While no significant difference was observed among groups at 24 h, the activity in group Eg remained significantly lower than that in group Cg from 48 h onwards (*p* < 0.05). At 96 h, the inhibitor composition effectively maintained enzyme activity at a much lower level compared to group Cg. These results demonstrate that the inhibitor treatment significantly suppressed the diesel-induced overactivation of autolytic enzymes in a time-dependent manner.

#### 3.2.2. Acetylcholinesterase (AchE) Activity

**Figure 4:**
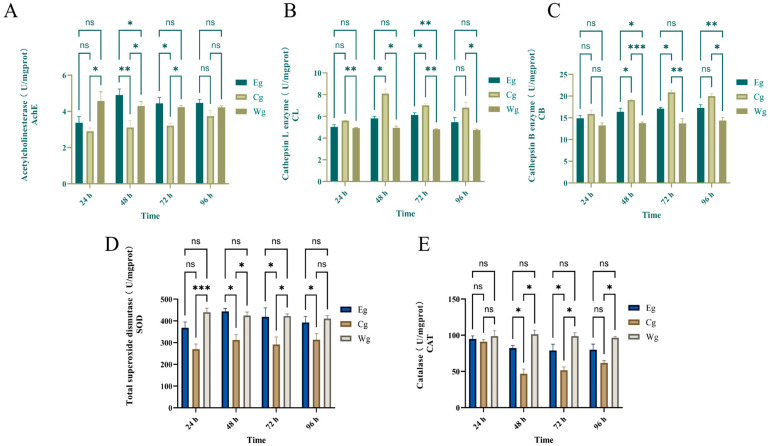
Effects of the inhibitor composition on body wall-related enzyme activities in *A. japonicus* exposed to No. 0 diesel oil. (**A**) acetylcholinesterase (AchE); (**B**) cathepsin L (CL); (**C**) cathepsin B (CB); (**D**) superoxide dismutase (SOD); (**E**) catalase (CAT). Note: “ns” indicates no significant difference. Significant differences are indicated by asterisks: * for (*p* < 0.05), ** for (*p* < 0.01), and *** for (*p* < 0.001).

As shown in Figure 4A, diesel exposure significantly suppressed AChE activity in group Cg. At 48 h, the AChE activity in Cg was significantly lower than that in Eg (*p* < 0.01) and Wg (*p* < 0.05), representing only 63.4% of the activity levels in group Eg. The inhibitor treatment (Eg) effectively counteracted this suppression; AChE activity in Eg remained significantly higher than in Cg from 48 h to 72 h (*p* < 0.05). By 96 h, no significant difference was observed between groups Eg and Wg, indicating that the inhibitor composition facilitated a robust recovery of neural signal transmission to near-normal levels.

#### 3.2.3. Cathepsin L (CL) Activity

As shown in Figure 4B, CL activity in group Cg increased significantly and peaked at 48 h (*p* < 0.05), remaining substantially higher than in group Eg despite a decline after 72 h. Group Eg showed a markedly smaller rise, maintaining only 71.6% of the Cg level at the 48 h peak. By 96 h, group Eg returned to a level comparable to group Wg, and no significant difference was observed between group Eg and group Cg. These results indicate that the inhibitor composition effectively mitigates the abnormal activation of CL during the peak stress period.

#### 3.2.4. Cathepsin B (CB) Activity

As shown in Figure 4C, CB activity in group Cg rose continuously from 24 h to 72 h. At 24 h, no significant difference was observed among the three groups. From 48 h to 72 h, group Cg stayed significantly higher than group Eg (*p* < 0.05). Specifically, at 48 h, CB activity in group Eg was 16.6% lower than in group Cg. By 96 h, group Eg remained 13.6% lower than group Cg but showed no significant difference. This suppression during the peak stress period confirms a stable inhibitory effect on CB activation.

#### 3.2.5. Total Superoxide Dismutase (SOD) Activity

As shown in Figure 4D, diesel exposure impaired the antioxidant capacity of group Cg, which maintained the lowest SOD activity among all groups at all time points. In contrast, the inhibitor significantly preserved SOD function from the onset. At 24 h, SOD activity in Eg was 1.29-fold that of Cg, and it continued to approach Wg levels. By 96 h, SOD activity in Eg was nearly identical to Wg, while Cg remained at only 79.9% of the Eg level (*p* < 0.05), indicating that the composition maintains the first line of antioxidant defense.

#### 3.2.6. Catalase (CAT) Activity

As shown in Figure 4E, CAT activity in all groups remained stable at 24 h with no significant differences. At 48 h, activity in group Cg dropped sharply to its lowest point, representing only 56.8% of the level observed in group Eg (*p* < 0.05). Although group Cg activity showed a slight recovery by 72 h, it still failed to reach the level of the inhibitor-treated group, remaining at only 67.7% of the group Eg value (*p* < 0.05). By 96 h, group Eg activity remained higher than in group Cg, but no significant difference was observed between the two groups.

### 3.3. Transcriptomic Results Analysis

#### 3.3.1. Quality of the Transcriptome Sequencing Data

Both Eg and Cg included three biological replicates (*n* = 3). After filtering raw reads, removing low-quality sequences, and examining sequencing error rates and GC content distribution, clean reads were obtained, as shown in Table 3. The clean ratios of all samples exceeded 94.5%, with Q20 > 95% and Q30 > 90%, indicating that the sequencing data were of high quality and suitable for subsequent analyses.

#### 3.3.2. Principal Component Analysis (PCA) Among Simples

**Figure 5:**
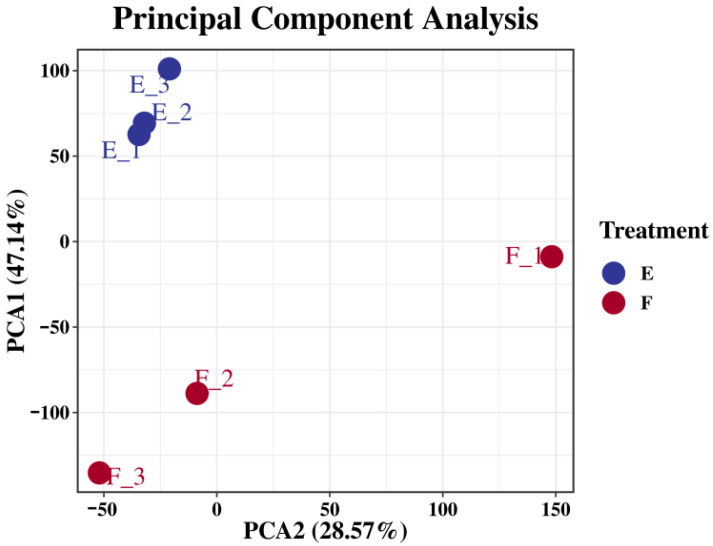
PCA results of treatment groups and the control group.

**Figure 6:**
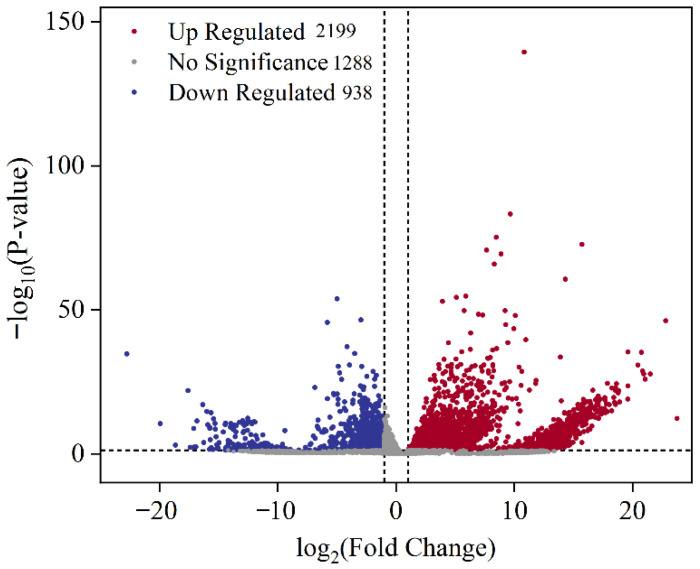
Volcano plot showing differentially expressed genes (DEGs) between groups Eg and Cg.

As shown in Figure 5, the PCA results showed partial separation between groups Eg and Cg, indicating a certain degree of specificity. Samples within the same group were clustered closely, demonstrating good intra-group consistency. The principal component analysis further confirmed that the sequencing data were suitable for differential gene expression analysis, and the results were reliable. As shown in Figure 6, compared with group Cg, a total of 3137 differentially expressed genes (DEGs) were identified in group Eg, including 2199 upregulated and 938 downregulated genes.

**Figure 7:**
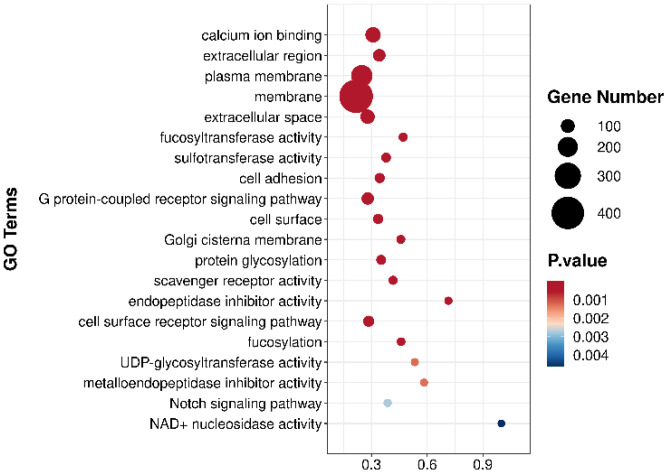
Gene Ontology (GO) enrichment analysis of differentially expressed genes (DEGs).

**Figure 8:**
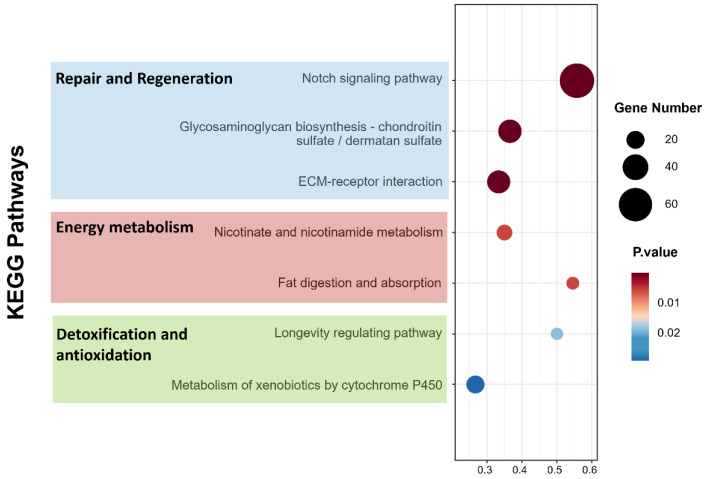
Kyoto Encyclopedia of Genes and Genomes (KEGG) pathway enrichment analysis of differentially expressed genes (DEGs).

To better understand the biological functions and interactions among the DEGs, all DEGs were annotated using Gene Ontology (GO) terms. The top 20 GO terms are presented in Figure 7. Subsequently, Kyoto Encyclopedia of Genes and Genomes (KEGG) pathway enrichment analysis was performed to identify the DEGs and biological pathways involved in the regulation of skin ulceration in *A. japonicus* (Figure 8). Through KEGG pathway analysis, genes closely associated with differential pathways were screened, and their upregulated or downregulated expression patterns are summarized in Table 4.

#### 3.3.3. GO Enrichment Analysis

To clarify the biological mechanisms underlying 0# diesel stress and further explore the functions of differentially expressed genes (DEGs), a GO functional enrichment analysis was conducted on 14,254 significantly DEGs (*p* < 0.05). These DEGs were enriched in 3887 GO terms, comprising 2475 Biological Processes (BPs), 932 Molecular Functions (MFs), and 479 Cellular Components (CCs). According to the top 20 GO terms shown in Figure 7, the enrichment highlights a systematic response to diesel stress. First, for biological processes, cell adhesion, G protein-coupled receptor signaling pathway, protein glycosylation, cell surface receptor signaling pathway, fucosylation, and Notch signaling pathway were prominently enriched. Second, regarding molecular functions, the response was characterized by the enrichment of calcium ion binding, fucosyltransferase activity, sulfotransferase activity, scavenger receptor activity, endopeptidase inhibitor activity, UDP-glycosyltransferase activity, and metalloendopeptidase inhibitor activity. Finally, for cellular components, the DEGs were primarily localized to the extracellular region, plasma membrane, membrane, extracellular space, cell surface, Golgi cisterna membrane, and NAD+ nucleosidase activity. This structured molecular response suggests that the sea cucumbers establish a multi-layered defense network to counteract 0# diesel stress.

#### 3.3.4. KEGG Pathway Enrichment Analysis

According to the results shown in Table 4, several metabolic pathways closely related to the skin ulceration process in *A. japonicus* contained significantly differentially expressed genes. KEGG enrichment analysis highlighted that the inhibitor composition significantly modulated three functional clusters of pathways to counteract diesel stress. First, for tissue structural integrity and regeneration, the Notch signaling and ECM–receptor interaction pathways showed marked activation. This was characterized by the significant upregulation of key genes including *HES-C* and fibrillin family members such as *fbn Ib* and *fbn1*. Second, regarding defensive mechanisms, pathways for xenobiotic metabolism including metabolism of xenobiotics by cytochrome P450 and antioxidant defense such as *Glutathione* metabolism were prominently enriched. These pathways exhibited a synergistic upregulation of *UGTs*, *CYP1A1*, and *GSTs*. Finally, energy metabolism pathways, particularly Nicotinate and nicotinamide metabolism along with glycerolipid metabolism, were significantly activated. These activations likely provided the necessary metabolic support for repair and detoxification processes. This structured molecular response suggests that the inhibitor composition establishes a multi-layered defense network rather than acting on a single target.

#### 3.3.5. qRT-PCR Validation

**Figure 9:**
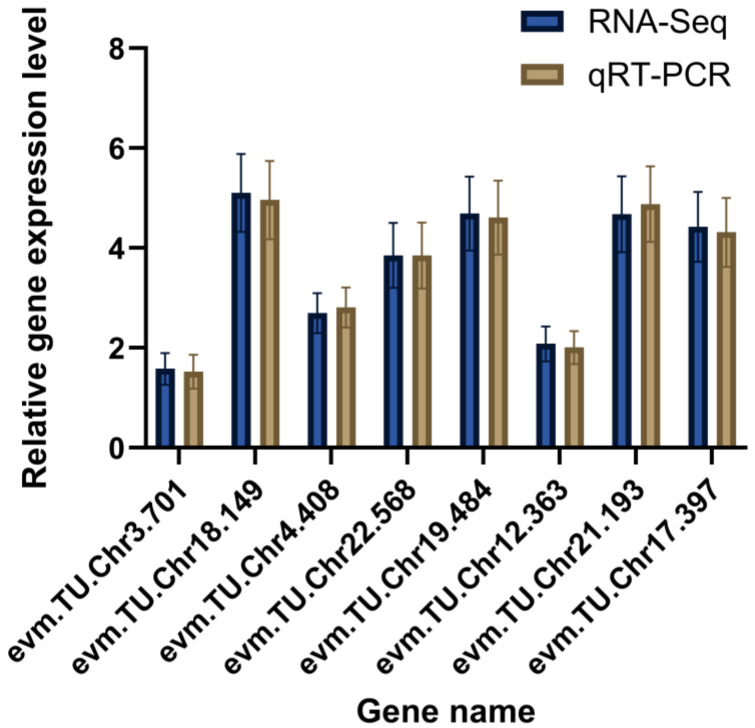
qRT-PCR validation of differentially expressed genes in *A. japonicus*.

We validated differential expression by qRT-PCR. Across the eight assayed genes, qRT-PCR estimates of relative abundance largely mirrored the directions observed in the RNA-seq data (Figure 9).

## 4. Discussion

### 4.1. Inhibitory Effects of the Inhibitor Composition on Skin Ulceration of A. japonicus Under No. 0 Diesel Oil Pollution Stress

The experimental results showed that the inhibitor composition significantly reduced diesel-induced skin ulceration in *A. japonicus*. At 96 h, the treated group (Eg) exhibited an ulceration area of 14.44 ± 1.79%, markedly lower than the diesel control (Cg, 33.19 ± 2.94%; *p* < 0.05). No ulceration was observed at 24 h in either group, indicating a delayed response to diesel stress, which aligns with previous reports [16,20]. While this protective effect mirrors results seen under UVA and *V. splendidus* stress [14,21], the degree of mitigation varied. This variation may suggest that the inhibitor targets conserved downstream pathological pathways rather than stressor-specific initial triggers. This hypothesis appears to align with the convergent pathology of skin ulceration syndrome (SUS) observed in various sea cucumber species, which potentially occurs regardless of biotic or abiotic origins [17]. Since tissue autolysis and apoptosis likely represent convergent endpoints, the inclusion of protease and caspase inhibitors might help block these final stages. Specifically, endogenous matrix metalloproteinases (MMPs) are thought to drive collagen degradation and autolysis in *A. japonicus* [22] while multiple caspase members may mediate stress-induced apoptosis [23]. Consequently, although diesel oil contains unique toxicants like aromatic hydrocarbons, the resulting degradation pathways seem to converge with those activated by other stressors, potentially rendering them susceptible to the same inhibitory strategy. These findings suggest a broad-spectrum potential for the composition in managing complex environmental stress.

### 4.2. Effects of the Inhibitor Composition on Enzyme Activity Changes in the Body Wall of A. japonicus Under No. 0 Diesel Oil Pollution Stress

The enzymatic activity shifts observed in this study reflect a deep physiological restructuring of *A. japonicus* during diesel-induced stress. The sustained rise in autolytic enzyme activity in group Cg, which peaked at 72 h, reveals a continuous activation of tissue degradation pathways. Autolytic enzymes are hydrolytic proteins that mediate intracellular protein breakdown and damaged tissue clearance [24]. However, their overactivation disrupts cell membrane integrity and accelerates skin autolysis. In group Cg, this “autolytic cascade” might be triggered by diesel-induced lysosomal instability. Lipophilic components in diesel oil may increase membrane permeability, possibly leading to the leakage of destructive proteases. In contrast, the inhibitor kept autolytic activity in group Eg significantly lower than in group Cg (e.g., 54.9% of Cg at 48 h). This suggests the inhibitor may stabilize lysosomal membranes, a mechanism also reported for natural chelators during sea cucumber autolysis regulation [21]. Such stabilization could prevent the premature release of cathepsin L (CL) and cathepsin B (CB), which might help break the positive feedback loop of tissue liquefaction [20]. These findings align with the inhibitor’s efficacy in UVA and *Vibrio* models and confirm that maintaining lysosomal stability is a universal strategy for preserving tissue integrity [14].

Diesel exposure suppressed AchE activity in group Cg, particularly during the 24–48 h period. This suppression likely disrupts acetylcholine hydrolysis, which impairs neuromuscular coordination [25] and metabolic balance [26] in *A. japonicus*. The inhibitor restored AchE activity in group Eg to near-Wg levels by 96 h. This recovery might mitigate neurotoxicity, perhaps by reducing PAH bioavailability or preventing PAH binding to the enzyme. These results are consistent with prior observations of stress-induced AchE disruption in sea cucumbers [26]. This neuroprotective effect complements structural protection, allowing the organism to better coordinate metabolic resources for repair rather than mere stress survival.

As lysosomal cysteine proteases, CL and CB play critical roles in tissue proteolysis under stress [27,28]. Their higher activation in group Cg indicates that diesel may increase lysosomal membrane permeability [10]. The suppression of these enzymes in group Eg demonstrates that the inhibitor effectively limits abnormal protease activation. This finding aligns with the role of cathepsin L in sea cucumber autolysis reported by Yan et al., further confirming that inhibiting protease overactivation is a vital mechanism for mitigating tissue degradation [28].

The reduced SOD activity in group Cg aligns with findings that oil pollution impairs the antioxidant capacity of *A. japonicus* [11]. SOD is the first line of defense against reactive oxygen species (ROS) [29], and its inhibition leads to lipid peroxidation and membrane damage [30]. The inhibitor maintained higher SOD activity in group Eg to help preserve radical scavenging efficiency. Furthermore, the increase in CAT activity at 96 h likely enhanced H_2_O_2_ decomposition [31]. This synergistic antioxidant effect mirrors Cai et al.’s observation that ascorbic acid alleviates autolysis via antioxidant enzyme activation [32].

Overall, the regulation of autolytic, neurofunctional, and antioxidant enzymes reflects a coordinated strategy to mitigate diesel-induced damage. By suppressing autolysis, restoring neural transmission, and enhancing oxidative defense, the inhibitor maintains cellular homeostasis. These results validate the multi-level physiological regulatory effects and broad-spectrum efficacy of the composition.

### 4.3. Transcriptomic Effects of the Inhibitor Composition on A. japonicus Under No. 0 Diesel Oil Pollution Stress

Previous studies have shown that No. 0 diesel oil contains multiple aromatic hydrocarbons that can either directly penetrate *A. japonicus* tissues and damage epidermal cell integrity or indirectly trigger autolysis through exogenous stimulation [33]. Under natural conditions, such as the Dalian Xingang oil spill, petroleum hydrocarbons can persist in sediments and impose long-term ecological burdens [34]. Evidence from in situ mesocosm experiments suggests that such spills drastically alter macrobenthic assemblages [35] potentially causing severe physiological stress in *A. japonicus*. This stress may manifest as intense oxidative damage and cellular apoptosis, particularly within the respiratory tree [9]. However, *A. japonicus* possesses strong tissue repair and regeneration capabilities. In this study, under No. 0 diesel oil pollution stress, the inhibitor composition significantly activated several signaling pathways related to tissue repair and cellular regeneration, including the Notch signaling pathway, Glycosaminoglycan biosynthesis–chondroitin sulfate/dermatan sulfate, and the ECM–receptor interaction pathway. Among them, the Notch signaling pathway is known to serve as a crucial regulator of tissue growth and regeneration [36]. The central role of this pathway in echinoderm tissue repair and cellular differentiation has been demonstrated in regeneration models, particularly during the critical stages of organ regrowth in *A. japonicus* [37]. Within this pathway, the Hairy and enhancer of split transcription factor C (*HES-C*) gene was markedly upregulated. As a member of the HES family, *HES-C* may inhibits the expression of pro-apoptotic genes [38]. The upregulation of *HES-C* induced by the inhibitor composition suggests that the treatment may promote cell survival and suppress apoptosis, thereby maintaining tissue structural stability during the early phase of chemical insult.

Simultaneously, genes associated with extracellular matrix (ECM) remodeling showed pronounced responses. Members of the fibrillin family (*fbn Ib*, *fbn1*) and several collagen-coding genes (such as *Collagen alpha-2*, *Collagen alpha-1 (IX)*, and *5 alpha fibrillar collagen*) were upregulated. Fibrillin family proteins may stabilize epithelial structure by interacting directly with cell-surface receptors or indirectly regulating other ECM-related proteins and cell adhesion molecules [39,40]. Collagen-encoding genes also play essential roles in maintaining ECM integrity [41]. The upregulation of these genes suggests that the inhibitor composition potentially reinforces the body wall’s physical barrier, leveraging the unique structural plasticity and defense capabilities of the echinoderm “adhesome” and its mutable collagenous tissues [42,43,44]. In contrast, several integrin family genes (*Integrin αP*, *Integrin β1-A*, and *Integrin α9-like*) were significantly downregulated. Integrins are key mediators of cell migration and signal transduction; previous research reported that β-integrin in *A. japonicus* mediates phagocytosis in response to *V. splendidus* infection [45]. Therefore, the downregulation of integrins observed in this study might reduce cell death and help stabilize tissue structures. It is also possible that inhibited integrin expression limits cell migration, potentially decreasing the likelihood of pollutant contact or further tissue injury [46]. Meanwhile, the *Zonadhesin* gene was markedly upregulated. Although typically involved in fertilization in mammals, Zonadhesin has also been reported to participate in cell adhesion in marine organisms [47]. Its upregulation in this study suggested enhanced epithelial cell junctions. The *A. japonicus* body wall contains two principal sulfated polysaccharides—sulfated fucans (SFs) and fucosylated chondroitin sulfates (FucCSs) [48]. In our study, the genes involved in sulfated polysaccharide biosynthesis (*CST15*, *CST14*, *CST11*, and *CST13*) were likewise upregulated. This pattern indicates that the inhibitor may enhance the sea cucumber’s capacity to repair its body wall by promoting the biosynthesis of structural glycans like FucCS. As FucCS is covalently associated with collagen fibrils [49], its increased synthesis likely strengthens the extracellular matrix scaffold, while its established role in accelerating tissue regeneration [50] further supports the recovery of body wall integrity under stress. Collectively, these results indicated that the inhibitor composition enhanced the regenerative capacity and structural homeostasis of *A. japonicus* body wall tissues at the molecular level by activating Notch signaling, promoting ECM remodeling and cell adhesion, and suppressing cell migration and apoptosis, thereby mitigating tissue damage induced by diesel exposure.

Detoxification processes represent one of the most important defense systems by which organisms resist environmental pollutants [51]. In this study, several genes belonging to the UDP-glucuronosyltransferase (*UGT*) family were significantly upregulated, suggesting that the inhibitor composition enhanced glucuronidation-based detoxification in *A. japonicus*. *UGTs* catalyze the conjugation of exogenous hydrophobic compounds with glucuronic acid, converting them into water-soluble conjugates for excretion and thereby reducing pollutant accumulation and cytotoxic risk. At the same time, the significant upregulation of Cytochrome P450 1A1 (*CYP1A1*) indicated that the inhibitor composition activated the cytochrome P450-based xenobiotic metabolism system. This system plays a central role in the oxidative metabolism of organic pollutants such as polycyclic aromatic hydrocarbons (PAHs) [52]. The induction of *CYP1A1* suggests that the inhibitor may have activated a coordinated multistage detoxification response. In this process, Phase I oxidation likely facilitates the efficient removal of diesel residues through collaborative biotransformation, a mechanism evidenced in marine invertebrates under chemical stress [53] and during major physiological shifts in *A. japonicus* [54].

Furthermore, oil-derived pollutants can induce excessive reactive oxygen species (ROS) generation, leading to oxidative stress, which can be counteracted by enzymatic and non-enzymatic antioxidant defense systems within the organism [55]. In group Eg, multiple antioxidant enzyme-encoding genes, including Glutathione S-transferase (*GST*), Microsomal *GST*3, and Glutathione S-transferase theta-1-like, were significantly upregulated and enriched in the Metabolism of xenobiotics by cytochrome P450 pathway. These gene products facilitate ROS removal and promote the conjugation and elimination of harmful metabolites, thereby mitigating oxidative damage [56]. Exposure to various stressors activates specific gene expression programs. Heat-shock proteins (HSPs) are key components of these responses and serve as important biomarkers of environmental stress in *A. japonicus* [57]. In this study, *hsp26* was significantly upregulated in group Cg, which is consistent with previous findings showing that heat shock proteins, including the small heat shock protein *hsp26*, are rapidly induced in *Apostichopus japonicus* in response to environmental stressors and petroleum-derived pollutants [58,59]. In contrast, *hsp26* was downregulated in group Eg. This suggests that the presence of the inhibitor composition effectively reduced stress levels and lessened dependence on an intense HSP response to maintain protein homeostasis. Collectively, these results demonstrated that the inhibitor composition established a multilayered molecular defense system by enhancing glucuronidation, activating the CYP450 detoxification system, increasing *GST* activity, and alleviating oxidative stress, thereby strengthening the detoxification and antioxidant capacities of *A. japonicus*.

The marked upregulation of energy metabolism-related pathways indicated that metabolic activity in *A. japonicus* was enhanced under No. 0 diesel oil pollution, supporting the increased energy demand for repair, defense, and detoxification processes. In marine invertebrates, environmental stress commonly disrupts energy homeostasis, leading to metabolic reallocation in which limited energetic resources are diverted from growth or reproduction toward essential survival-related processes such as maintenance, defense, and detoxification [60,61]. In particular, the Nicotinate and nicotinamide metabolism pathway was significantly activated following inhibitor composition treatment, with key genes *NAD kinase isoform 1* and *ADP-ribosyl cyclase isoform X1* being upregulated. This pathway involves the metabolism of NAD^+^ and NADP^+^, which play central roles in energy supply and redox balance [56]. The elevated utilization of NAD^+^ may represent a key feature of body wall energy metabolism under diesel stress, as NAD^+^ plays a central role in cellular bioenergetics, redox homeostasis, and DNA repair, thereby supporting the high-energy demands associated with cellular repair and stress adaptation [62,63]. This metabolic enhancement reflected an adaptive energy mechanism in *A. japonicus*. Meanwhile, the activation of the fat digestion and absorption pathway further indicated enhanced lipid metabolism. Lipase-related genes, including *inactive pancreatic lipase-related protein 1* and *pancreatic lipase-related protein 3-like (PNLIPRP)*, were significantly upregulated in group Eg. These genes promote lipid hydrolysis and provide substrates for energy metabolism [64]. Although marine invertebrates lack a typical pancreas, previous studies have shown that lipase-like proteins are expressed in some tissues of invertebrate species [65], suggesting that these genes may serve similar roles in *A. japonicus* body wall tissues. Overall, diesel exposure substantially increased the energy demands of *A. japonicus*, and the inhibitor composition potentially enhanced energy supply by activating nicotinamide metabolism and lipid degradation pathways, thereby providing the necessary metabolic support for tissue repair, regeneration, and detoxification.

**Figure 10:**
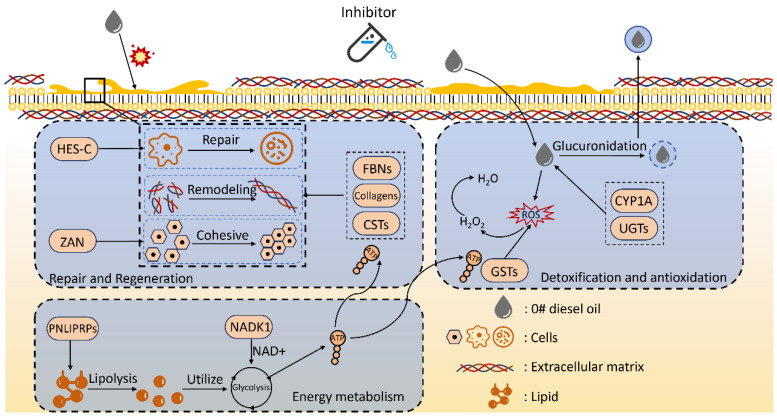
Transcriptomic mechanism of action.

In summary, the inhibitor composition achieved systemic protective effects under No. 0 diesel oil pollution through multilayered regulation. First, by activating Notch signaling, promoting ECM remodeling, and enhancing cell adhesion, it maintained tissue regenerative capacity and structural homeostasis. Second, by activating key enzyme systems such as *UGT*, CYP450, and *GST*, it significantly enhanced detoxification and antioxidant defenses. Third, by strengthening nicotinamide metabolism and lipid degradation pathways, it improved energy supply to sustain repair and defense responses. Together, these molecular responses revealed the broad-spectrum inhibitory effects and comprehensive protective mechanisms of the inhibitor composition across multiple physiological systems. This study not only verified the effectiveness of the inhibitor composition in mitigating diesel-induced damage, but also elucidated its mechanistic pathways in energy metabolism, detoxification defense, and tissue repair. It provides new theoretical evidence for a better understanding of its physiological regulatory effects (Figure 10).

## 5. Conclusions

This study demonstrates that the inhibitor composition (Nafamostat mesylate, Ilomastat, and Emricasan) significantly alleviates No. 0 diesel oil-induced skin ulceration in *Apostichopus japonicus*, reducing the lesion area from 33.19% (diesel-exposed controls) to 14.44% (treated group) at 96 h. The composition suppressed overactivation of autolytic enzymes (cathepsin L and B), restored acetylcholinesterase activity, and maintained antioxidant enzyme (SOD and CAT) function under diesel stress. Transcriptomic analysis revealed coordinated regulation of tissue repair (Notch signaling, ECM-receptor interaction), detoxification (*CYP1A1*, *UGTs*, *GSTs*), and energy metabolism pathways. These findings suggest that the inhibitor composition exerts protective effects through multi-level molecular mechanisms and may have broad-spectrum potential against diverse environmental stressors in sea cucumber aquaculture.

## Figures and Tables

**Table 1:** Gene and primer design table.

Gene ID	Primer Sequence
*evm.TU.Chr3.701*	F: GCTCTAGGGGTTACAATGGC R: AACCGTTCCTTGCAATCCTT
*evm.TU.Chr18.149*	F: CTTGGCTTGTATTGGCGTTG R: CTCATCCTCGGGTATGGGAT
*evm.TU.Chr4.408*	F: GAGAGCTGGTGTTGCATGTA R: GACGGTATCAAACGCCTTCT
*evm.TU.Chr22.568*	F: CGAACTGGAGATTGATGGGG R: TTCCCGACAAAGAAATCCCC
*evm.TU.Chr19.484*	F: TATGTGGCTGTCCCGAAATG R: GCTCTCCCGTGTAGTATCCT
*evm.TU.Chr12.363*	F: AGAGGGCGAGGCTATTACAT R: TGTTTTCTCCCTGCGTTCAA
*evm.TU.Chr21.193*	F: TGCTGTTTGGTGCTCTTCAT R: CACGTCACATGGTTCTTTGC
*evm.TU.Chr17.397*	F: ACATCGCCAAGAACATACCC R: AAGAGCCTTCCAACAACCAG
*NADH*	F: GTCCTACGACCCAATCTGGA R: ATGAGCCTTGGTTACGTTGG

**Table 2:** Inhibitory effects of the inhibitor composition on the skin ulceration area ratio (%) of *A. japonicus* induced by No. 0 diesel oil.

Group	24 h	48 h	72 h	96 h
Eg	0%	4.67 ± 0.49% *	8.96 ± 0.79% *	14.44 ± 1.79% *
	−	+	+	++
Cg	0%	8.36 ± 0.98% *	14.27 ± 1.95% *	33.19 ± 2.94% *
	−	+	++	+++
Wg	0%	0%	0%	0%
	−	−	−	−

Note: Data are presented as mean ± SD (*n* = 3). Less than 1% of the skin is affected: −; 1–10% of the skin is affected: +; 10–30% of the skin is affected: ++; 30–50% of the skin is affected: +++. * indicates a significant difference (*p* < 0.05).

**Table 3:** Summary of the sequencing data.

Sample	Raw Data	Clean Data	Clean Ratio	Q20 (%)	Q30 (%)	GC Content (%)
E_1	37,206,718	35,381,702	95.09	99.24	90.95	40
E_2	38,234,368	36,430,978	95.28	99.20	90.56	40
E_3	43,079,998	40,877,354	94.89	99.16	90.39	40
F_1	42,949,182	40,848,914	95.11	99.25	91.14	41
F_2	37,653,366	35,850,178	95.21	99.25	91.37	40
F_3	34,278,442	32,396,616	94.51	99.17	90.73	40

**Table 4:** Differentially expressed genes (DEGs) and their corresponding metabolic pathway information.

Gene Name	KEGG Pathway ID	EC Number	Description	Trend
*evm.TU.Chr12.225*	03460/04330	/	Hairy and enhancer of split transcription factor C	up
*evm.TU.Chr11.954*	04330/04371/04658/04919/05200/05206/05224/05165/01522	/	Fibropellin-1	up
*evm.TU.Chr3.701*	04512	/	Collagen alpha-1(IX) chain	up
*evm.TU.Chr18.149*	00532	2.8.2.33	Carbohydrate sulfotransferase 15	up
*evm.TU.Chr4.408*	00590/00591	1.14.14.1/ 1.14.14.73	Cytochrome P450 2J2-like	up
*evm.TU.Chr22.568*	00590/00480/00980/00982	5.3.99.2/ 2.5.1.18	Glutathione S-transferase 5 isoform X2	up
*evm.TU.Chr19.484*	00480/00980/00982/00983	2.5.1.18	Microsomal *GST*3	up
*evm.TU.Chr12.363*	00760	2.7.1.23	NAD kinase isoform 1	up
*evm.TU.Chr21.193*	00760	3.2.2.6/ 2.4.99.20	ADP-ribosyl cyclase isoform X1	up
*evm.TU.Chr17.397*	00561/04972/04975	3.1.1.3	Inactive pancreatic lipase-related protein 1	up
*evm.TU.Chr7.576*	00561/04972/04975	3.1.1.3	Putative pancreatic lipase-related protein 3-like	up

**Figure A1:**
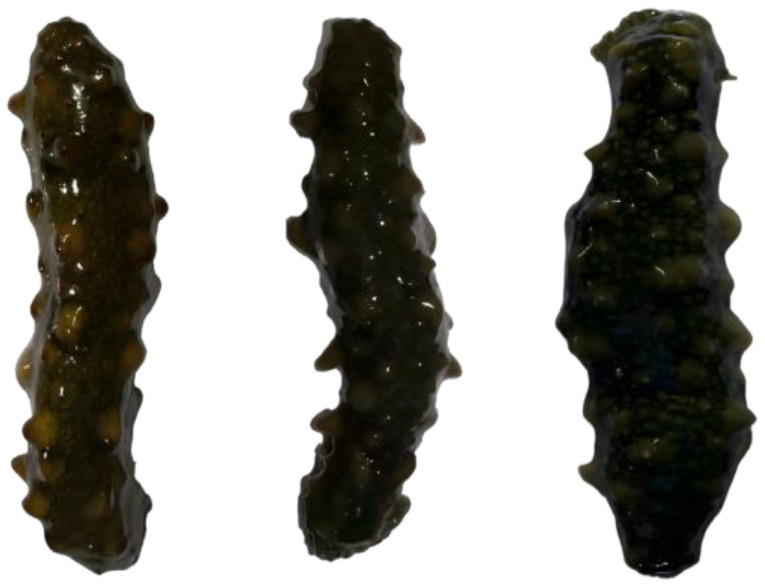
Morphological status of Group Eg at 24 h.

**Figure A2:**
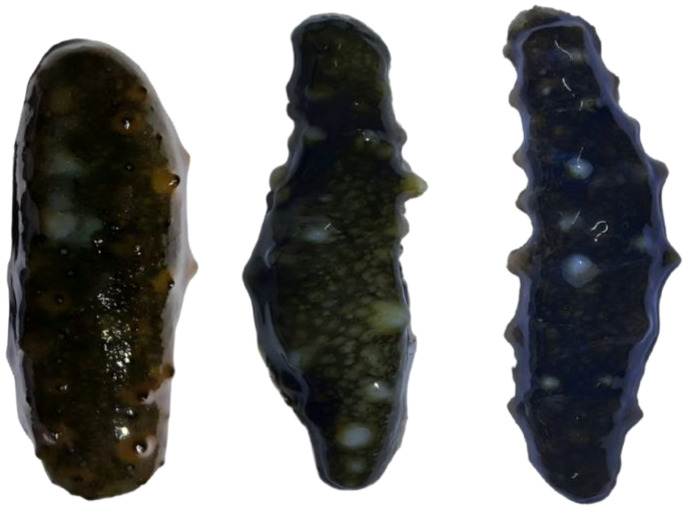
Morphological status of Group Eg at 48 h.

**Figure A3:**
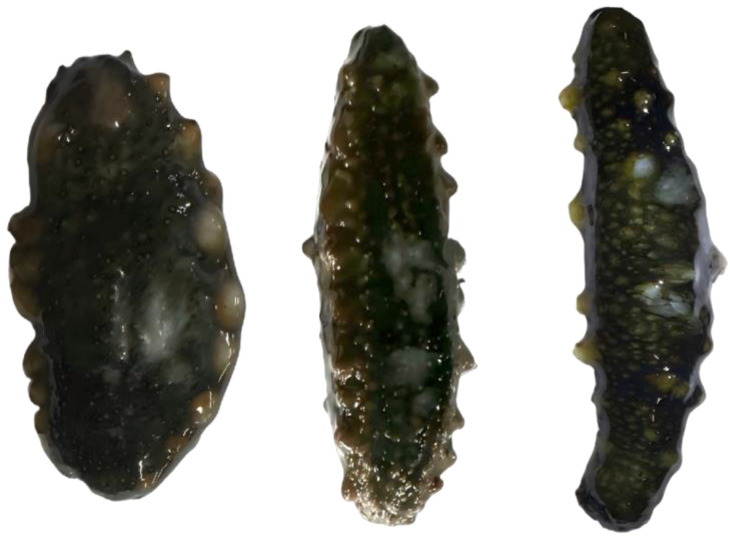
Morphological status of Group Eg at 72 h.

**Figure A4:**
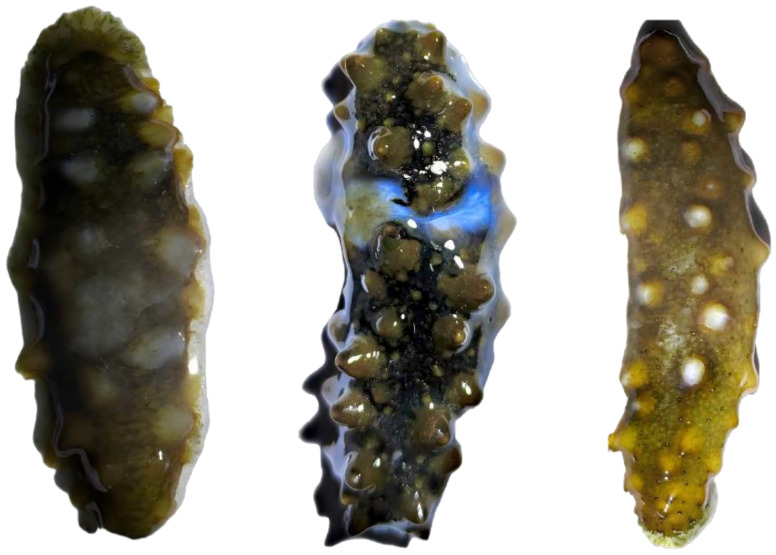
Morphological status of Group Eg at 96 h.

**Figure A5:**
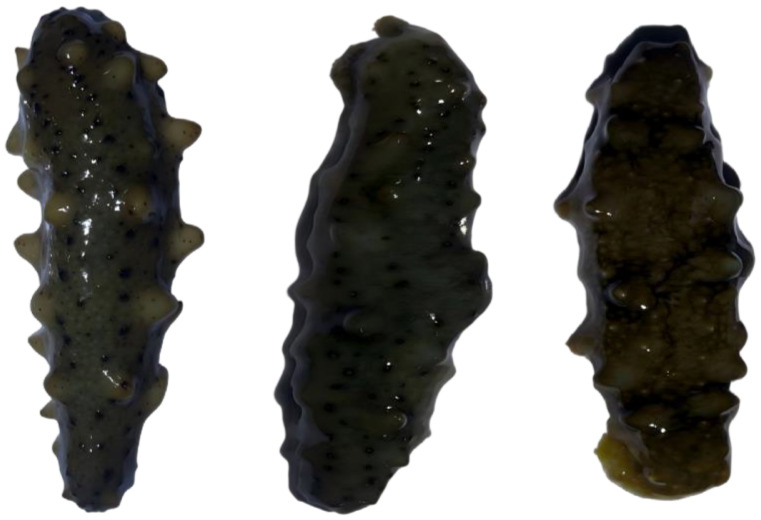
Morphological status of Group Cg at 24 h.

**Figure A6:**
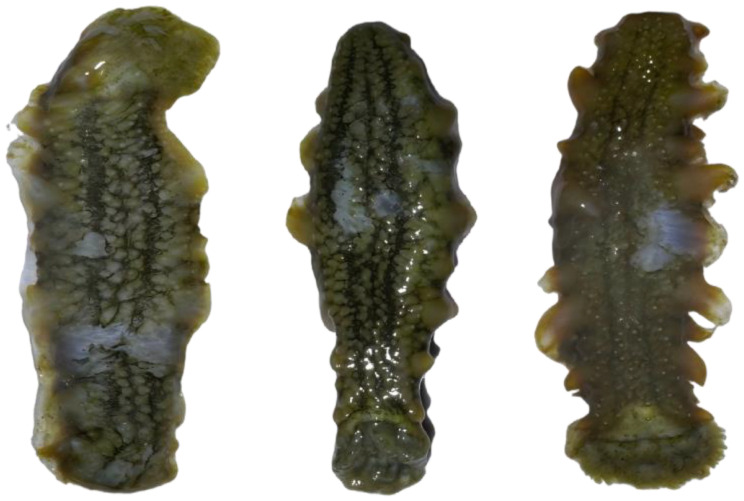
Morphological status of Group Cg at 48 h.

**Figure A7:**
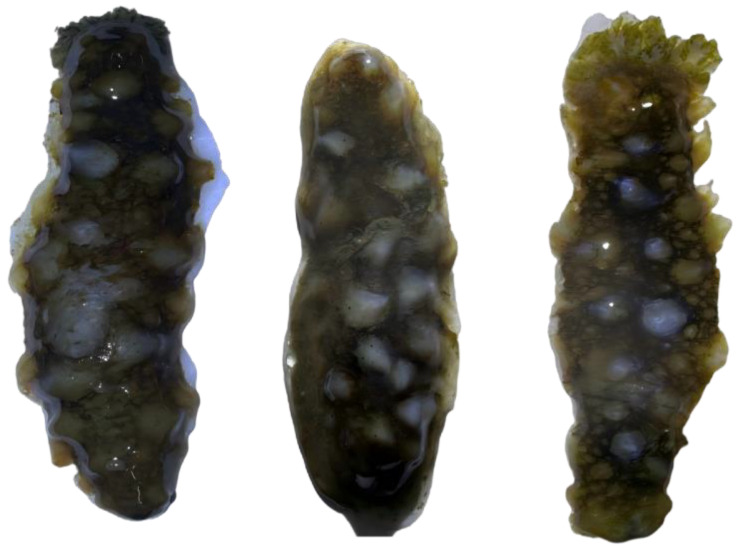
Morphological status of Group Cg at 72 h.

**Figure A8:**
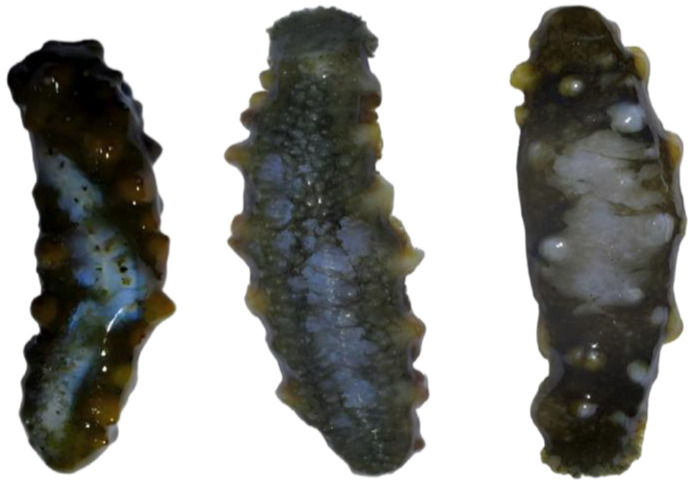
Morphological status of Group Cg at 96 h.

**Figure A9:**
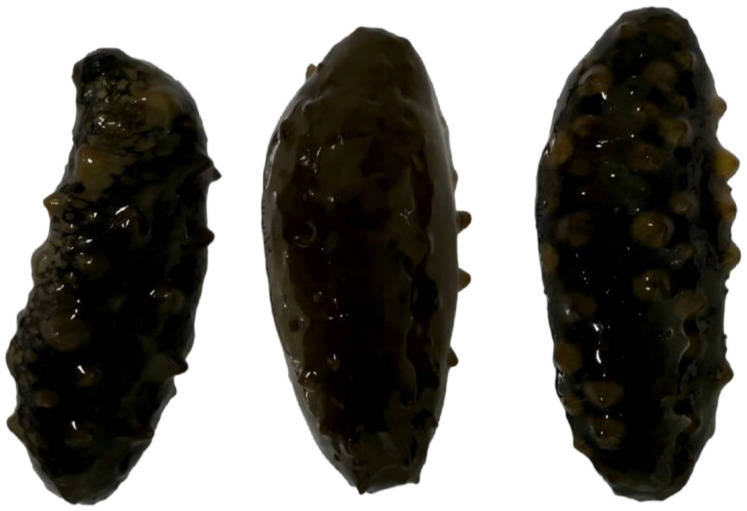
Morphological status of Group Wg at 24 h.

**Figure A10:**
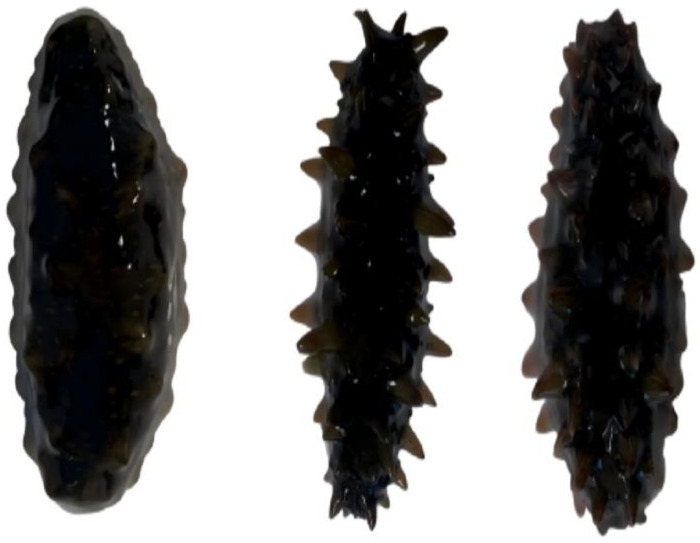
Morphological status of Group Wg at 48 h.

**Figure A11:**
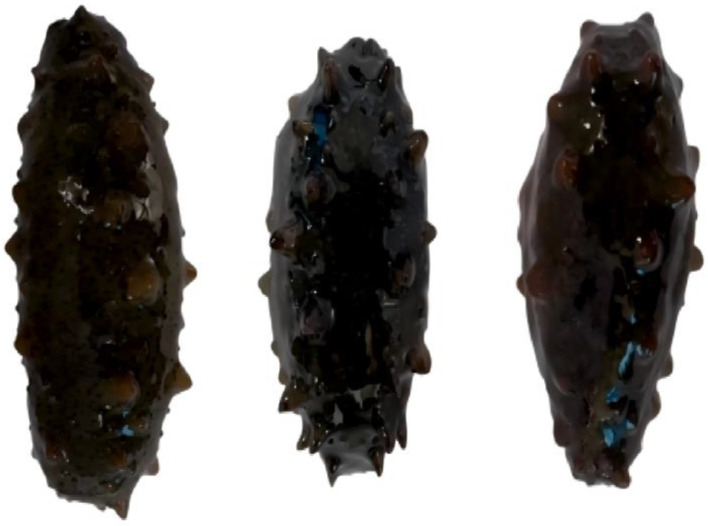
Morphological status of Group Wg at 72 h.

**Figure A12:**
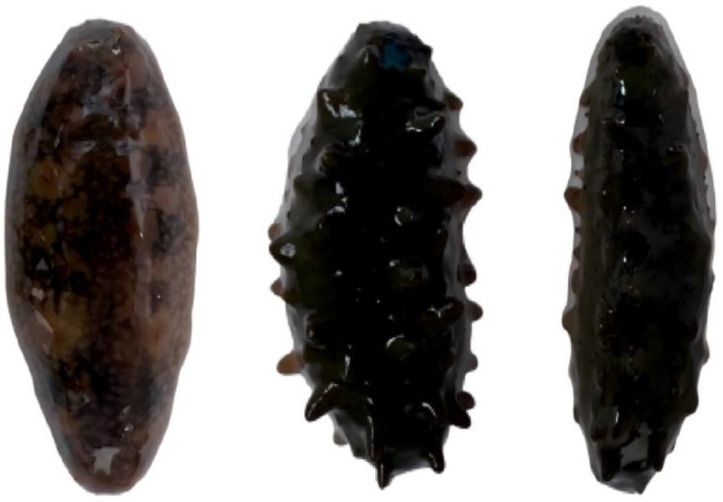
Morphological status of Group Wg at 96 h.

## Data Availability

All relevant data are presented within the paper.
